# Epoxy pre-polymers as new and effective materials for corrosion inhibition of carbon steel in acidic medium: Computational and experimental studies

**DOI:** 10.1038/s41598-019-48284-0

**Published:** 2019-08-12

**Authors:** Omar Dagdag, Zaki Safi, Rachid Hsissou, Hamid Erramli, Mehdi El Bouchti, Nuha Wazzan, Lei Guo, Chandrabhan Verma, E. E. Ebenso, Ahmed El Harfi

**Affiliations:** 1 Laboratory of Agroresources, Polymers and Process Engineering (LAPPE), Department of Chemistry, Faculty of Science, Ibn Tofail University, BP 133, 14000 Kenitra, Morocco; 2 Al Azhar University-Gaza, Chemistry Department, Faculty of Science, P.O Box 1277, Gaza, Palestine; 3 Laboratory of Materials, Electrochemistry and Environment, Department of Chemistry, Faculty of Sciences, Ibn Tofail University, Kenitra, Morocco; 4 Higher School of Textile and Clothing Industries, Laboratory REMTEX, BP 7731, Oulfa, Casablanca Morocco; 5 King Abdulaziz University, Chemistry Department, Faculty of Science, P.O Box 42805, Jeddah, 21589 Saudi Arabia; 6 School of Materials and Chemical Engineering, Tongren University, Tongren, 554300 China; 7 Material Science Innovation & Modelling (MaSIM) Research Focus Area, Faculty of Natural and Agricultural Sciences, North-West University, Private Bag X2046, Mmabatho, 2735 South Africa; 8 Department of Chemistry, Faculty of Natural and Agricultural Sciences, School of Chemical and Physical Sciences, North-West University, Private Bag X2046, Mmabatho, 2735 South Africa

**Keywords:** Materials science, Engineering

## Abstract

Present study is designed for the synthesis, characterization and corrosion inhibition behavior of two diamine aromatic epoxy pre-polymers (DAEPs) namely, N^1^,N^1^,N^2^,N^2^-tetrakis (oxiran-2-ylmethyl) benzene-1,2-diamine (DAEP1) and 4-methyl-N^1^,N^1^,N^2^,N^2^-tetrakis (oxiran-2-ylmethyl) benzene-1,2-diamine (DAEP2) for carbon steel corrosion in acidic medium. Synthesized DAEPs were characterized using spectral (Nuclear magnetic resonance (^1^H NMR) and Fourier transform infrared-attenuated total reflection (FTIR-ATR)) techniques. Viscosity studies carried out at four different temperatures (20–80 °C) increase in temperature causes significant reduction in their viscosities. The anticorrosive properties of DAEPs differing in the nature of substituents, for carbon steel corrosion in 1 M HCl solution was evaluated using several experimental and computational techniques. Both experimental and computational studies showed that inhibitor (DAEP2) that contains electron releasing methyl (-CH_3_) showed higher protectiveness as compared to the inhibitor (DAEP1) without substituent (-H). Electrochemical results demonstrate that DAEPs act as reasonably good inhibitors for carbon steel in 1 M HCl medium and their effectiveness followed the sequence: DAEP2 (92.9%) > DAEP1 (91.7%). The PDP results show that the diamine aromatic epoxy pre-polymers molecules (DAEPs) act as mixed type inhibitors. Electrochemical study was also supported using scanning electron microscopy (SEM) method were significant improvement in the surface morphology of inhibited (by DAEPs) metallic specimens was obtained. Results derived from computational density functional theory (DFT) and molecular dynamics (MD) simulationsand studies were consistent with the experimental results derived from SEM, EIS and PDP electrochemical studies. Adsorption of the DAEPs obeyed the Langmuir adsorption isotherm model.

## Introduction

Corrosion of steel alloys is one of the most important safety and economic anxiety for many industries including oil-gas and petroleum industries. Carbon steel is one of the frequently used steel based materials for numerous many applications in several industries because of its excellent mechanical power and relatively low cost. However, it is highly susceptible to corrosion during several industrial processes where metallic components undergo corrosive dissolution by aggressive acidic attack. Hydrochloric acid is one of the most utterly used acids for these processes due to the fact of its relatively low cost, aggressive nature, efficiency in comparison to other mineral acids. However, the continuous utilization of acids in these repetitive processes may induce corrosion of steel materials and then deterioration of the industrial tools^1–3^. The addition of corrosion inhibitor into the acid is used to reduce the corrosive attack of acid by adsorbing themselves onto the steel surface and prevents the direct contact of acid with the casing and tubing steel^4,5^. The corrosion inhibitors can adsorb at the interfaces of metals and electrolytes and form protective surface covering with the aid of polar functional groups constituted by heteroatoms (N, P, O, and S) as well as through the π-electrons of functional groups and aromatic rings. Among the inhibitors which are effective in acid solutions there are nitrogen containing compounds (amines, amides, diamines)^6–10^. The computational studies present informations in relation to the effectiveness of the metal-inhibitor interactions along with the orientation of the inhibitor molecule(s) on the metallic surface. Employment of the DFT in corrosion inhibition of metallic surface in acidic medium in the presence of organic inhibitors is also gaining recent attention. With DFT-based studies, it became promising to gain detail about the organic film electronic features prevailing its adhesion manner with regards to substrate^11–15^. Apart from DFT study, computational study being carried out using MD simulations give some useful parameters through which metal-inhibitor(s) interactions and orientation of the inhibitors at meta-electrolyte interface can be derived^16,17^.

Epoxy pre-polymers are multifunctional macromolecular matrices that are widely used in large amounts as heavy-duty anticorrosion coatings due to its outstanding adhesion to many substrates, high strength, corrosion resistances and excellent chemical resistance properties^18,19^. Novelty of the present study lying on the fact that DAEPs are used as corrosion inhibitors for the first time therefore use of this type of compounds as corrosion inhibitors should be explored. Outcome of the study suggested that inhibitor containing methyl substituent showed higher effectiveness than the inhibitor without any substituent. Therefore, present study will help in the designing of the effective corrosion inhibitors for future studies. The selection of diamine aromatic epoxy pre-polymers (DAEPs) as corrosion inhibitors in present investigation is also based on the facts that they are associated extensive conjugation in the form of multiple (double) bonds of aromatic rings and non-bonding electrons of heteroatoms (oxygen and nitrogen) that can offer strong interactions with the metallic surface. Further, DAEPs are also associated with highly reactive four epoxy rings in their molecular structures that easily undergoes acid catalyzed ring opening reaction and get converted into hydroxyl group (-OH) that can further enhance their adsorption ability on metallic surface as well as solubility in the polar electrolytic medium of 1 M HCl. In present study, we synthesized, characterized and demonstrated the anticorrosive property the two diamine aromatic epoxy pre-polymers (DAEPs) namely,N^1^,N^1^,N^2^,N^2^-tetrakis (oxiran-2-ylmethyl) benzene-1,2-diamine (DAEP1) and 4-methyl-N^1^,N^1^,N^2^,N^2^-tetrakis (oxiran-2-ylmethyl) benzene-1,2-diamine (DAEP2) on carbon steel corrosion in acidic medium. In the first part of our present study, we demonstrated the viscosity and viscoelastic measurements of both the investigated (DAEPs) molecules and outcomes of the study showed DAEPs at 20 °C behave as Newtonian liquids and heightening of the temperature reduced their viscosity. Numerous viscoelastic properties including storage modulus (*G*′), loss modulus (*G*″) and complex viscosity (η*) as a function of angular frequency at different temperatures (20–60 °C) is also evaluated. Both DAEPs are evaluated for their anticorrosive behavior on carbon steel corrosion in 1 M HCl solution using PDP, EIS, SEM, DFT and MD methods. More so, diamine aromatic epoxy pre-polymers contain several electron rich sites in the form of atoms other than carbon (N and O) and π-electrons by way of which they can adsorb effectually and obstruct corrosion thereafter. Both investigated compounds act as good corrosion inhibitors. Thermodynamic parameters for the steel corrosion in 1 M HCl solution with and without diamine aromatic epoxy pre-polymer DAEP2 at its 10^−3^ M concentration were evaluated at various temperatures (298–328 K). The adsorption and inhibition behavior of diamine aromatic epoxy pre-polymers DAEPs were supported by DFT and MD computational techniques.

## Experimental

### Materials

**Figure 1:**
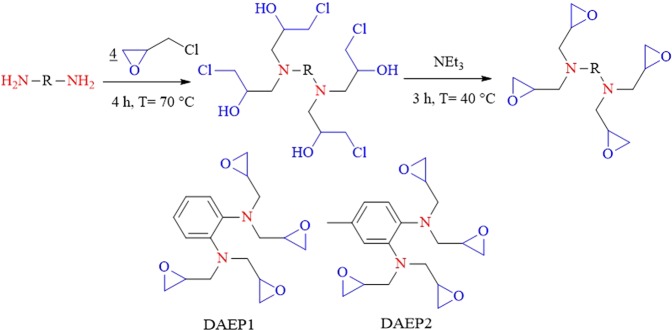
Schematic outline for the synthesis of two diamine aromatic epoxy pre-polymers DAEP1 and DAEP2.

The chemicals and materials used in this work such as 1,2-Diaminobenzene (≥99%), 4-Methyl-1,2-phenylenediamine (≥98%), epichlorohydrin (99%) and triethylamine (≥99.5%) were purchased from Aldrich chemical company. Steel alloy having composition described in our previous study^20–22^ were used as working (test) material. Preparation of the electrolyte and metallic specimens can be found elsewhere^23^. The DAEPs was synthesized by the reaction of diamine with epichlorohydrin in the presence of triethylamine (NEt_3_) as per the course of action reported in the literature^24,25^. Schematic diagram for the preparation of the diamine aromatic epoxy pre-polymers (DAEPs) is shown in Fig. 1. Informations related to the synthesized DAEP sare given in Figs SI1 and SI2 and Table SI2. In brief, aromatic diamines (10^−2^ mol) were dissolved and in 10 mL of ethanol (10 mL) in two-neckRB flask. In the above reaction mixture, 2.5 mL of epichlorohydrin (2.5 mL) was added slowly with continuous stirring. Resultant mixture was allowed to worm around 70 °C for 4 h. The resultant reaction mixture was again allowed to stir at 40 °C for 3 h, after the addition of 3 mL of triethylamine (NEt_3_). Viscous solution of desired resins was obtained by evaporating residual solvents using a rotary evaporator. Synthesized inhibitors were characterized using spectral techniques. Infrared (IR) spectra were registered with a Bruker Fourier transform infrared (FTIR) using the FT-IR-attenuated total reflection technique. Following parameters were used: resolution 4 cm^−1^, spectral range 500–4000 cm^−1^, number of scans 128. ^1^H NMR spectra were recorded on a Bruker AVANCE 300 MHz instrument with DMSO-d_6_ as solvent and (CH_3_)_4_Si as an internal standard. All ^1^H-NMR experiments are reported in δ units.

### Viscosity and viscoelastic measurements

The viscosity and viscoelastic measurements of the two synthesized DAEPs were studied using Haake Mars rheometer having 25 mm diameter parallel plate geometry with a 0.5 mm gap. The viscosity measurements were carried out at a shear rate ranged between 0.01 and 150 s^−1^ at different temperatures ranging from 20 to 80 °C. The dynamic rheology measurements (dynamic viscoelastic parameters such as the elastic *G*′, *G*″ and η*) as a function amplitude sweep 100–0.1 rad/s angular frequency were conducted at different temperatures ranging from 20–60 °C.

### Electrochemical measurements

The electrochemical behavior of DAEPs was studied using Potentiostat (BioLogic SP-200 instrument as described in our earlier reports^26,27^. Stock solution of the inhibitors (DAEP1 and DAEP2) was prepared by dissolving them in small amount (nearly 2 mL) of isopropyl alcohol followed by the addition of tested electrolyte. Sample preparation and instrumental procedure for electrochemical studies was same as reported previously^26,27^. In order to get more accuracy and reproducibility of experimental data, the electrochemical studies were performed triply at each tested concentration of the DAEP1 and DAEP2 and mean values are reported. Inhibition effect of the DAEPswas evaluated using Eqs (1) and (2).

1 $${\eta }_{EIS}\, \% =\frac{{R}_{ct}-{R}_{ct}^{0}}{{R}_{ct}}\times 100$$

2 $${\eta }_{{\rm{PDP}}}\,( \% )=(1-\frac{{i}_{{\rm{corr}}}}{{i}_{{\rm{corr}}}^{0}})\times 100$$

where, *R*_ct_^0^ and *R*_ct_ represent the charge transfer resistances values without and with DAEPs, respectively. Whereas, *i*^0^_corr_ and *i*_corr_ are the current densities for steel corrosion without and with the DAEPs, respectively.

### SEM characterization

For the surface study using SEM method, cleaned metallic specimens were allowed to dip and corrode for 12 h in 1 M HCl solution with and without DAEPs. After that metallic specimens were taken out bathed with sanitised water, and dried out in vacuity. The surfaces of the inhibited (by DAEPs) and uninhibited metallic specimens were inspected using a S3000H, Hitachi-Field Emission SEM instrument operated at 20 kV.

### Computational details

For DFT study, both DAEP1 and DAEP2 were geometrically optimized using B3LYP/6-31 + G(d, p) level^28,29^ in both gas phase and in aqueous solution as described elsewhere^30^. All calculations were performed using the G09 suite program^31^. Harmonic vibrational frequency study was employed to substantiate that all the structures obtained are read without imaginary functions (negative functions.) Since the experimental data were executed in acidic aqueous solution; the calculations accounted for solvent effect by applying. Visual inspections were performed using the GaussView program (version 5.0.8)^32^ and Chemcraft program version 1.8 (build 489) (Chemcraft-graphical software for visualization of quantum chemistry computations were performed for the Visual inspections of the outputs.

### Molecular dynamics simulations

MD simulations study was performed using Forcite module of Materials Studio 8.0 program developed by BIOVIA Inc. as described earlier^33–37^. A simulation box of 1.98 nm × 1.98 nm × 4.01 nm size consisting of periodic boundary conditions was selected for the simulations. The box consisted of a lower Fe slab and an upper solvent layer (containing 500 water molecules and one inhibitor molecule). For the iron substrate, Fe(110) was selected as explored surface since that it has a density packed structure and is the most stable. The Fe(110) exterior was modelled with a six-layer of 64 iron atoms in each *i*. *e*.(8 × 8) unit cell.

## Results and Discussion

### Viscosity measurement

**Figure 2:**
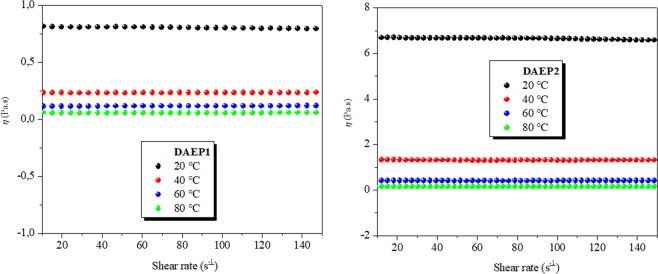
Viscosity measured as a function of shear for diamine aromatic epoxy pre-polymers DAEP1 and DAEP2 at different temperature.

The viscosities of DAEPs were restrained as a purpose of shear rate at different temperatures range 20–80 °C as given in Fig. 2. The evolutions of the viscosity for diamine aromatic epoxy pre-polymers DAEP1 and DAEP2 with temperature permitted the estimation of the viscosity activation energy. The activation energy (*E*_a_ (kJ/mol)) of the diamine aromatic epoxy pre-polymers were calculated by Eq. (3) ^38–40^.

3 $$\eta (T)={\eta }_{0}\exp (\frac{{E}_{a}}{RT})$$

where Viscosity (*η*) (Pa.s), *η*_0_ is a constant, *R* the gas constant (8.314 j/mol.K) and *T* is temperature in K.

Arrhenius plots for the viscosity were given in Fig. SI3. Values of *E*_a_ for diamine aromatic epoxy pre-polymers were estimated by calculating the slope of Ln (*η*) vs. 1/T.

The activation energies for DAEP1 and DAEP2 are 38.36 kJmol^−1^ and 57.91 kJmol^−1^, respectively. The results show that, the *E*_a_ for DAEP2 is higher than DAEP1 which can be attributed to the steric (electronic) repulsion between hydrophobic methyl (-CH_3_) substituent and epoxy cycle. Availability of the -CH_3_ in the molecular structure of DAEP2 enhances the bulkiness because of its high size as compared to the size of hydrogen of DAEP1. Apart from this, because of electron donating ability methyl substituent enhances the electron density over the aromatic ring which in turn affects the bulkiness of the entire molecule. It is important mention that the higher the activation energy value is related with higher viscosity^23^.

### Dynamic rheology measurements

**Figure 3:**
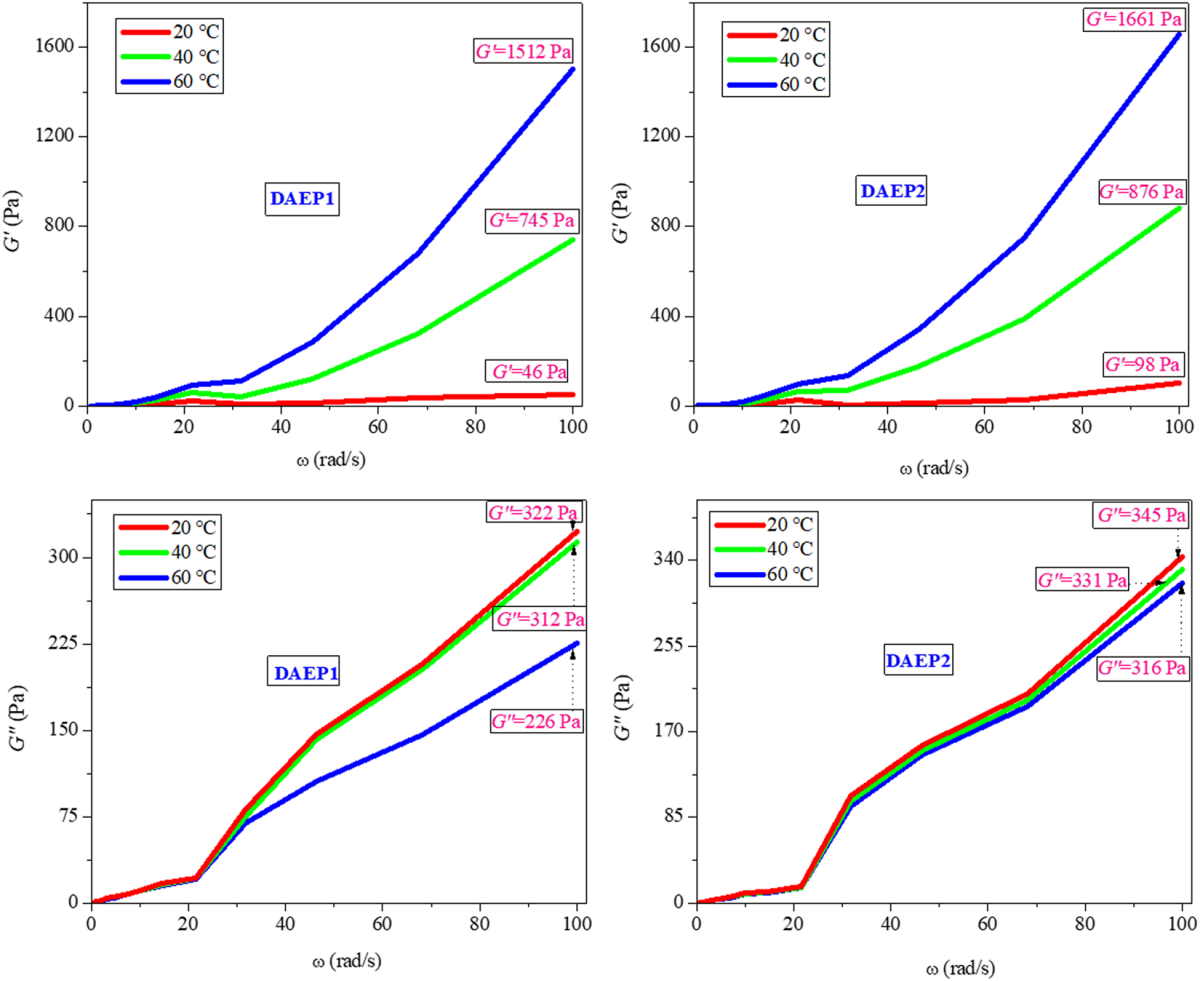
Changes of *G*′ and *G*″ as functions of frequency for DAEP1 and DAEP2 at different temperatures.

The dynamic rheology studies were conducted for DAEP1 and DAEP2 and some dynamic rheological parameters, such as *G*′, *G*″ and η* derived at different temperatures. The dynamic rheological studies reveal the informations about the structures and dynamics of investigated system (DAEPs). Figure 3 shows the *G*′ and *G*″ curves for the DAEPs. There is a well dependence of the dynamic modulus on the angular frequency as the *G*′ and *G*″ increases with the increasing the angular frequency. Larson^41^ illustrated that *G*′ and *G*″ values give information about prototypical solid like and liquid-like materials/properties. Generally, Liquid-like behavior is expected for *G*′ < *G*″ cases, whereas and solid-like behavior is expected for just inverse case (*G*′ > *G*″). From the Fig. 3, we can see that for DAEP1, *G*′ and *G*″ values are 46 Pa and 322 Pa, respectively, whereas for DAEP2*G*′ and *G*″ values are 98 Pa and 345 Pa, respectively which suggests that DAEPs showed the property of a liquid-like at 20 °C (*G*″ > *G*′). However, careful inspection of the results showed that at 40 °C, for DAEP1, *G*′ and *G*″ values are 745 Pa and 312 Pa, respectively, whereas for DAEP2 *G*′ and *G*″ values are 876 Pa and 331 Pa, respectively. Whereas, at 60 °C, DAEP1, *G*′ and *G*″ values are 1512 Pa and 226 Pa, respectively, whereas for DAEP2 *G*′ and *G*″ values are 1661 Pa and 316 Pa, respectively. For temperatures between 40 and 60 °C, the elastic modulus (*G*′), which was higher in magnitude than the loss modulus (*G*″) for both DAEPs showed the property of a solid state (*G*′ > *G*″). In order to avoid solid state property of the investigated molecules it is essential to store them at low temperature (T < 20 °C).

**Figure 4:**
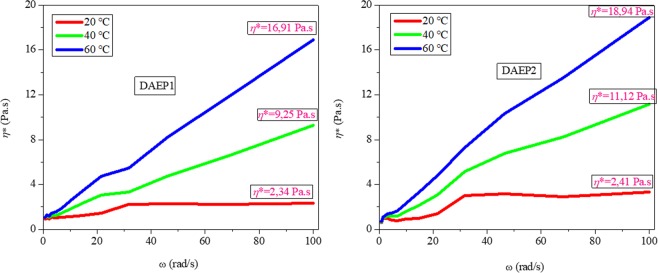
Changes complex viscosity as functions of frequency for DAEP1 and DAEP2 at different temperatures.

Figure 4 illustrates the complex viscosity (η*) as function angular frequency for both studied DAEPs at different temperature (20, 40 and 60 °C). The dynamic viscosity reaches a constant value of 2.34 Pa.s for DAEP1 that was 3.41 Pa.s for DAEP2 at 20 °C. Complex viscosity is characteristic of a Newtonian fluid behavior for DAEPs. It is also clear from the figure that viscosity does not depend on the angular frequency at 20 °C. At 40 °C, the dynamic viscosities reach to 9.25 and 11.12 Pa.s for DAEP1 and DAEP2, respectively. At 60 °C, dynamic viscosities further increased to 16.91 Pa.s and 18.94 Pa.s for DAEP1 and DAEP2, respectively. It is also noticeable that dynamic viscosity is temperature-dependent phenomenon. The increase in the dynamic viscosities of DAEPs with increasing the angular frequency suggests that the DAEPs show non-Newtonian rheological behavior of pseudo plastic typeat temperatures 40 and 60 °C.

### PDP study

**Figure 5:**
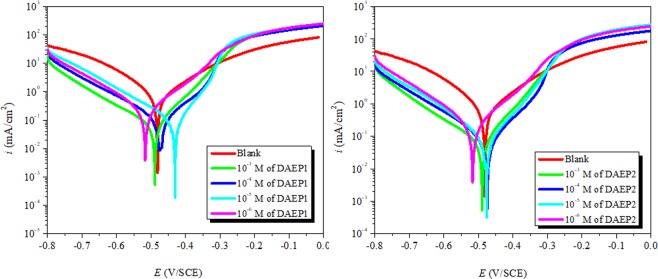
PDP curves for carbon steel corrosion in 1 M HCl solution in the absence and presence of different concentrations of DAEP1 and DAEP2.

The PDP curves for carbon steel dissolution without and with different concentrations of the DAEPs were measured to gather the information about kinetics of the anodic oxidative metallic dissolution and cathodic reductive hydrogen evolution.Fig. 5 shows the PDP curves without and with different concentrations of the diamine aromatic epoxy pre-polymers (DAEPs) and parameters are presented in Table 1.

**Table 1:** Potentiodynamic polarization parameters (±SD) for carbon steel corrosion in 1 M HCl solution in the absence and presence of different concentrations of DAEP1 and DAEP2.

Inh	*C* (M)	*E*_corr_ (mV/SCE)	*i*_corr_ (µA/cm^2^)	*β*_a_ (mV/dec)	−*β*_c_ (mV/dec)	*η*%
Blank	—	−473.80	916.6 (±1.78)	163.6 (±1.10)	155.0 (±1.33)	—
DAEP1	10^−3^ 10^−4^ 10^−5^ 10^−6^	−489.32 −473.58 −429.96 −516.72	075.40 (±0.36) 133.67 (±0.57) 172.98 (±0.85) 250.88 (±0.93)	149.7 (±0.87) 139.8 (±0.95) 133.9 (±0.91) 172.7 (±1.02)	151.3 (±1.79) 168.7 (±1.68) 168.2 (±1.67) 177.6 (±1.71)	91.7 85.4 81.2 72.6
DAEP2	10^−3^ 10^−4^ 10^−5^ 10^−6^	−489.28 −474.57 −476.71 −516.57	070.52 (±0.23) 089.45 (±0.28) 145.78 (±0.45) 212.64 (±0.87)	084.9 (±0.65) 067.7 (±0.42) 136.8 (±0.89) 111.4 (±0.77)	158.0 (±1.24) 106.1 (±1.02) 165.7 (±1.56) 134.7 (±1.45)	92.3 90.0 84.0 76.8

The values (Table 1) of cathodic Tafel lines, *β*_c_, show a slight change with increasing inhibitor concentration, indicating the influence of the DAEPs derivative on the kinetics of hydrogen evolution. This may probably be due to a diffusion or barrier effect. The values of the slopes of the anodic Tafel lines, *β*_a_, was noticeably changed which proposes the role of inhibitors molecules adsorption on the active anodic sites on the iron dissolution mechanism. The anomalous behavior of anodic curves is related to the metal surface passivation as a result of inhibitor film deposition. It can also be noted that very little change in the values of *E*_corr_ of the inhibited Tafel curves were observed with respect to the *E*_corr_ of black which suggests that DAEPs are mixed type corrosion inhibitors^42–44^. The inhibitory efficacies of the tested molecules followed the order: DAEP2 (92.3%) > DAEP1 (91.7%). The higher protectiveness of the DAEP2 as compared to DAEP1 can be attributed due to the presence of additional methyl substituent in the molecular structure of DAEP2 that enhances the electron density over the aromatic ring and thereby enhances the probability of the metal-inhibitor (DAEP2) interactions. The reasonably high inhibition effectiveness of the DAEP1 and DAEP2 can be attributed to the presence of several heteroatoms (N and O) that offer non-bonding electrons and aromatic rings that offer π-electrons for metal-inhibitor interactions. Apart from the metal-inhibitors (DAEPs) interactions, extensive presence of heteroatoms in the form of polar functional group(s) enhances the solubility of the investigated inhibitor molecules^6,23^. Results showed that DAEP 2 is a better corrosion inhibitor as compared to the DAEP1. It can be concluded that presence of methyl group (in DAPE2) can enhances the electron density over the active sites involving in the metal-inhibitor interactions. More so, after the adsorption of DAEP2 over the metallic surface, methyl group can enhance its protection efficiency by forming the hydrophobic surface film over the metallic surface. Therefore, methyl group can enhance the protection efficiency of DAEP2 as compared to the DAEP1 either by enhancing the electron density over the active sites because of its electron donating ability or by forming the hydrophobic surface films or by the combination of both.

### EIS study

**Figure 6:**
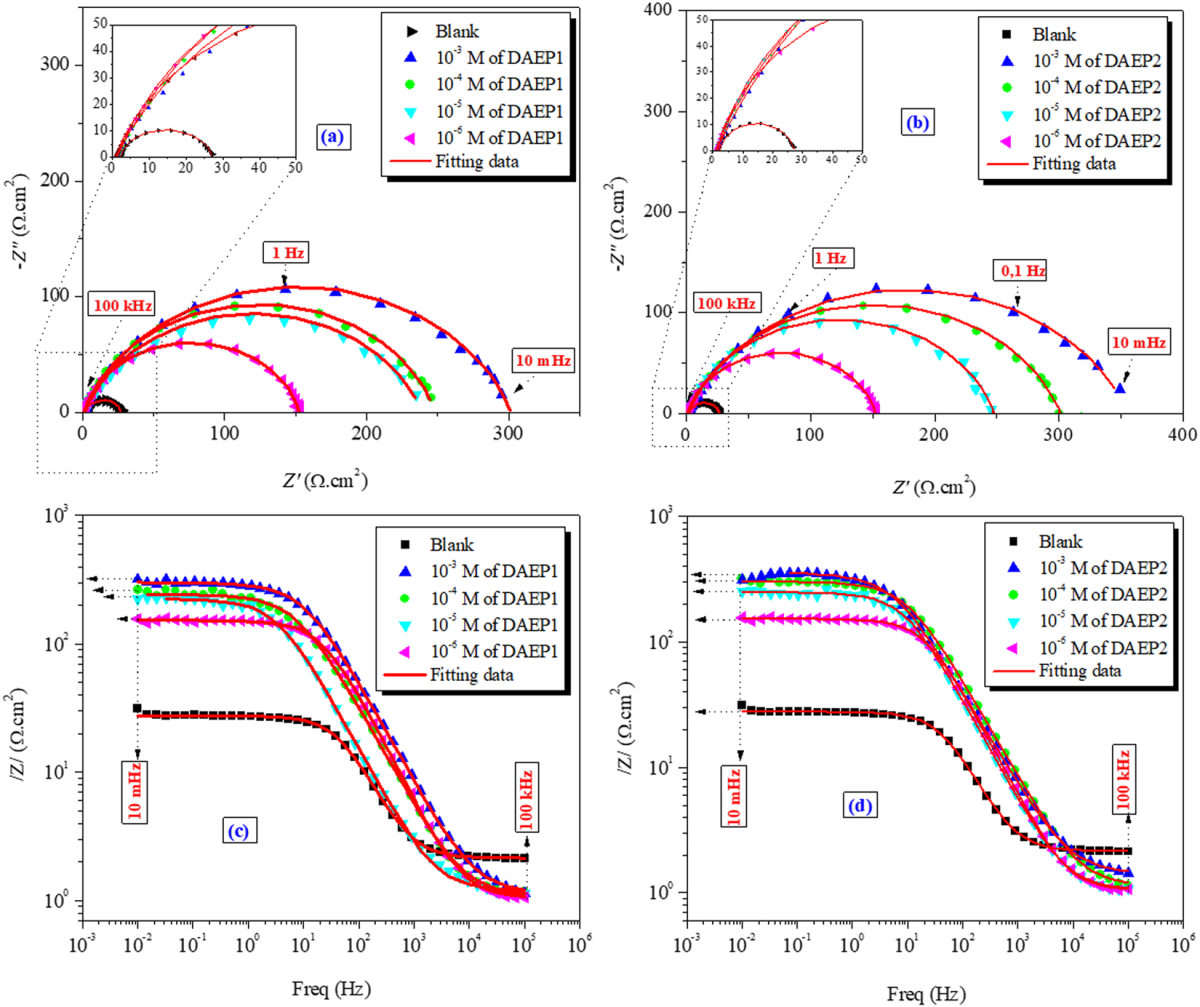
Nyquist and Bode diagrams for carbon steel in 1 M HCl solution in the absence and presence of different concentrations of DAEP1 and DAEP2.

The Nyquistand Bode plots for carbon steel corrosion in 1 M HCl are presented in Fig. 6. It is easy to notice that Nyquist curves present a single capacitive loop over at frequency range from 10 mHz to 100 kHz, which is usually related to charge transfer phenomenon^45^. The capacitive plots are semicircles (imperfect circle), this is accredited to frequency spreading as a consequence of inhomogeneous behavior of electrode surface^46^. Increase in the diameter of semicircle on addition of DAEP1 and DAEP2 indicates that charge transfer phenomenon retorted or slow down due to adsorption of DAEPs at the interface of metal-electrolyte. Moreover, increase in diameter of the Nyquist plots is consistent with the concentration of DAEPs. Equivalent circuit model (shown in Fig. SI4) was used for fitting of Nyquist plots and calculation of EIS indices. The CPE is defined as the below Eq. (4) ^47^:

4 $${Z}_{CPE}=\frac{1}{{Y}_{0}{(j\omega )}^{\alpha }}$$

In the above equation, all symbols have their usual meaning^48^. The capacitance values (*C*_dl_) is calculated using Eq. (5) ^49,50^:

5 $${C}_{dl}={Y}_{0}{({\omega }_{max})}^{n-1}$$

As can be seen from Table 2, the *R*_ct_ values are enhancing with increasing the inhibitor concentration that can be ascribed to the formation of adsorption layer on the steel surface. The adsorbed inhibitors (DAEPs) films actas barrier for charge-transfer at the electrode surface. Similar to PDP results, EIS results showed that anticorrosive property of DAEP2 is relatively higher than inhibition property of DAEP1which clearly indicates that presence of electron releasing methyl group favor the metal-inhibitor(DAEPs) interactions^51^. Moreover, the *C*_dl_ values diminishon enhancing the concentration for the DAEPs. This results from the increase in the thickness of the protective layer and/or the decrease in local dielectric constant of the film^52^.

**Table 2:** Electrochemical impedance spectroscopy parameters (±SD) for carbon steel corrosion in 1 M HCl solution in the absence and presence of different concentrations of DAEP1 and DAEP2.

Inh	*C* (M)	*R*_s_ (Ω. cm^2^)	*R*_ct_ (Ω. cm^2^)	*C*_dl_ (mF/cm^2^)	*η*%	χ^2^
Blank	—	2.15 (±0.03)	25 (±1.52)	4.6 (±0.04)	—	0.056
DAEP1	10^−3^ 10^−4^ 10^−5^ 10^−6^	1.12 (±0.01) 1.07 (±0.01) 1.18 (±0.02) 1.04 (±0.03)	300.4 (±3.36) 242.0 (±2.54) 224.0 (±2.32) 151.6 (±1.76)	0.15 (±0.01) 0.18 (±0.02) 0.35 (±0.02) 0.96 (±0.03)	91.7 89.7 88.8 83.5	0.109 0.358 0.251 0.218
DAEP2	10^−3^ 10^−4^ 10^−5^ 10^−6^	1.41 (±0.02) 1.13 (±0.01) 1.05 (±0.03) 1.04 (±0.01)	354.9 (±4.55) 300.4 (±3.23) 246.8 (±2.75) 151.1 (±1.96)	0.17 (±0.01) 0.12 (±0.01) 0.14 (±0.02) 0.26 (±0.02)	92.9 91.6 89.8 83.4	0.062 0.109 0.247 0.274

### Morphological analysis

**Figure 7:**
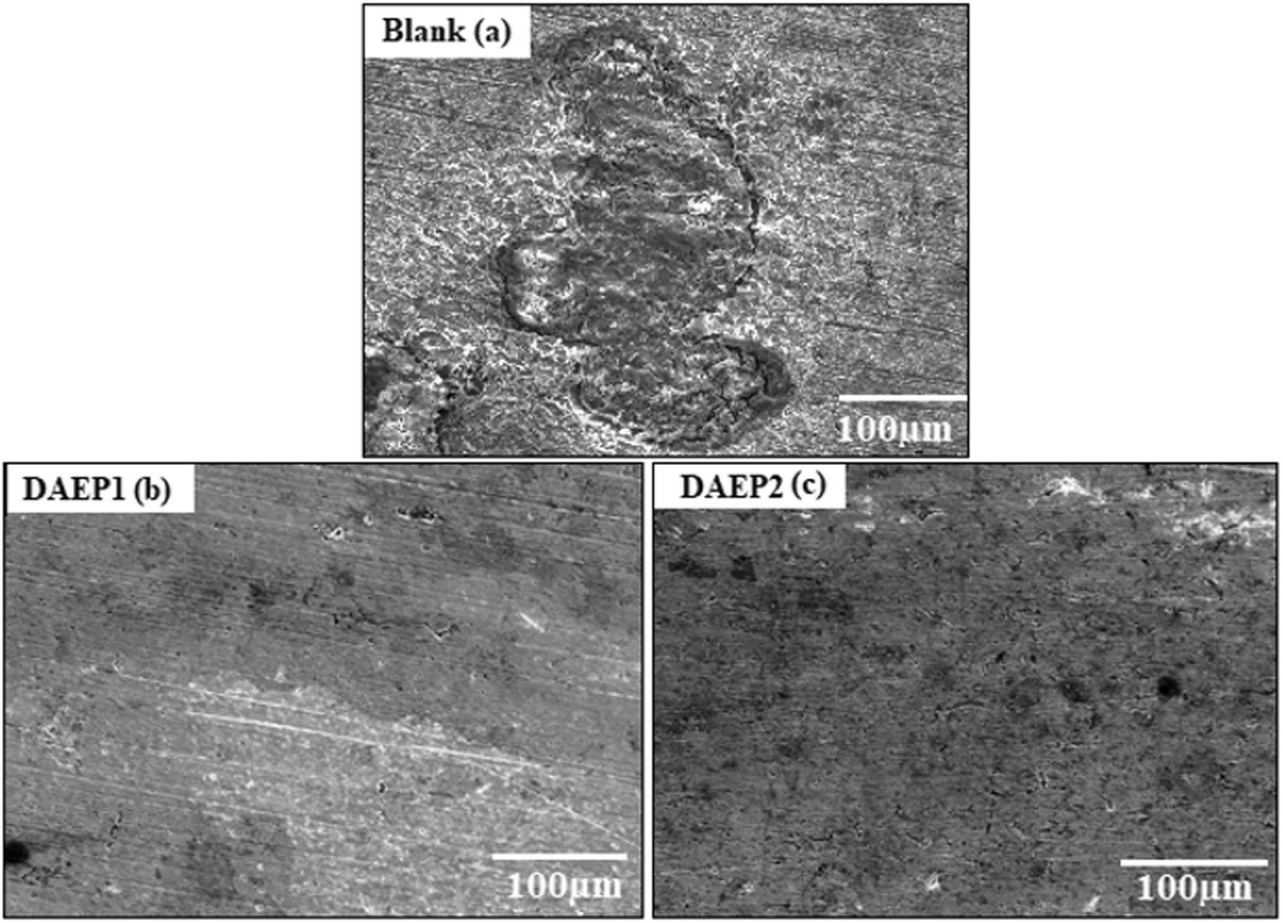
SEM micrographs of low carbon steel after 12 h of immersion in 1 M HCl solution: (**a**) without inhibitor, (**b**) and (**c**) with 10^−3^ M of DAEP1 and DAEP2, respectively.

The surface microstructure of the steel with the two diamine aromatic epoxy pre-polymers and in the absence of DAEP1 and DAEP2 was evaluated by SEM, and obtained micrographs are presented in Fig. 7(a–c). SEM observations taken after 12 h immersion time clearly show the difference between the three steel surfaces with and without DAEP1 and DAEP2. In Fig. 7a, the SEM micrograph for carbon steel after immersion in corrosive medium revealed that the metallic surface was damaged due to aggressive attack (from 1 M HCl solution), resulting in rapid corrosion. Figure 7b,c show the steel surface that was immersed for the same time interval in the acid solution containing 10^−3^ M concentration of DAEP1 and DAEP2, respectively. As can be seen, the surfaces damage has diminished due to the protective effects of DAEP1 (Fig. 7b) and DAEP2 (Fig. 7c), and particularly prominent in the case of the DAEP2 (Fig. 7c).

### Adsorption studies

Metal-inhibitors (DAEPs) interactions can be most appropriately explained with the help of isotherm model. In present study, adsorption of the DAEP1 and DAEP2 obeyed the Langmuir isotherm model that can be presented as follows (6)^53^.

6 $$\frac{{C}_{inh}}{\theta }=\frac{1}{{K}_{ads}}+{C}_{inh}$$

In above equation, all symbols have their usual meaning. Calculated values of *K*_ads_ were derived from intercept of the Langmuir isotherm model (i.e. *C*_*i*nh_ and *C*_*i*nh_/θ) shown in Fig. SI5. Values of the Δ*G*_ads_ were calculated using Eq. (7) ^54^:

7 $${K}_{ads}=\frac{1}{55.5}exp(\frac{-{\rm{\Delta }}{G}_{ads}}{RT})$$

Generally, high positive value of *K*_ads_ and high negative value of ΔG_ads_ are associated with strong metal-inhibitor interactions^55,56^. The higher values of *K*_ads_ for DAEP2 indicate that it has stronger absorption ability than DAEP1 (Table SI3). The calculated ΔG_ads_ values for diamine aromatic epoxy pre-polymersis superior to −40 kJ/mol, indicating strongly interaction of DAEP1 and DAEP2 onto the metallic surface (Table SI3)^57^. Moreover, ΔG_ads_ values for diamine aromatic epoxy (DAEPs) of these pre-polymersmolecules decreases in the order ΔG_ads_ (DAEP2) > ΔG_ads_ (DAEP1).

### Effect of temperature

PDP curves were recorded at different temperatures (298, 308, 318 and 328 K.) for carbon steel corrosion in 1 M HCl without and with DAEP1 and DAEP2 and derived results are presented in Fig. SI6. Results showed that protection efficiencyof DAEP1 and DAEP2 decreases on elevating the temperature (Table SI4). Therefore, increasing the temperature increases the corrosion current density from 75 to 923 μA. cm^−2^ for DAEP1, and from 70 to 501 μA. cm^−2^ for DAEP2, respectively. This result can be explaining by desorption of inhibitor molecules from carbon steel surface^6,23^.

### Theoretical studies

#### Quantum chemical calculation

**Figure 8:**
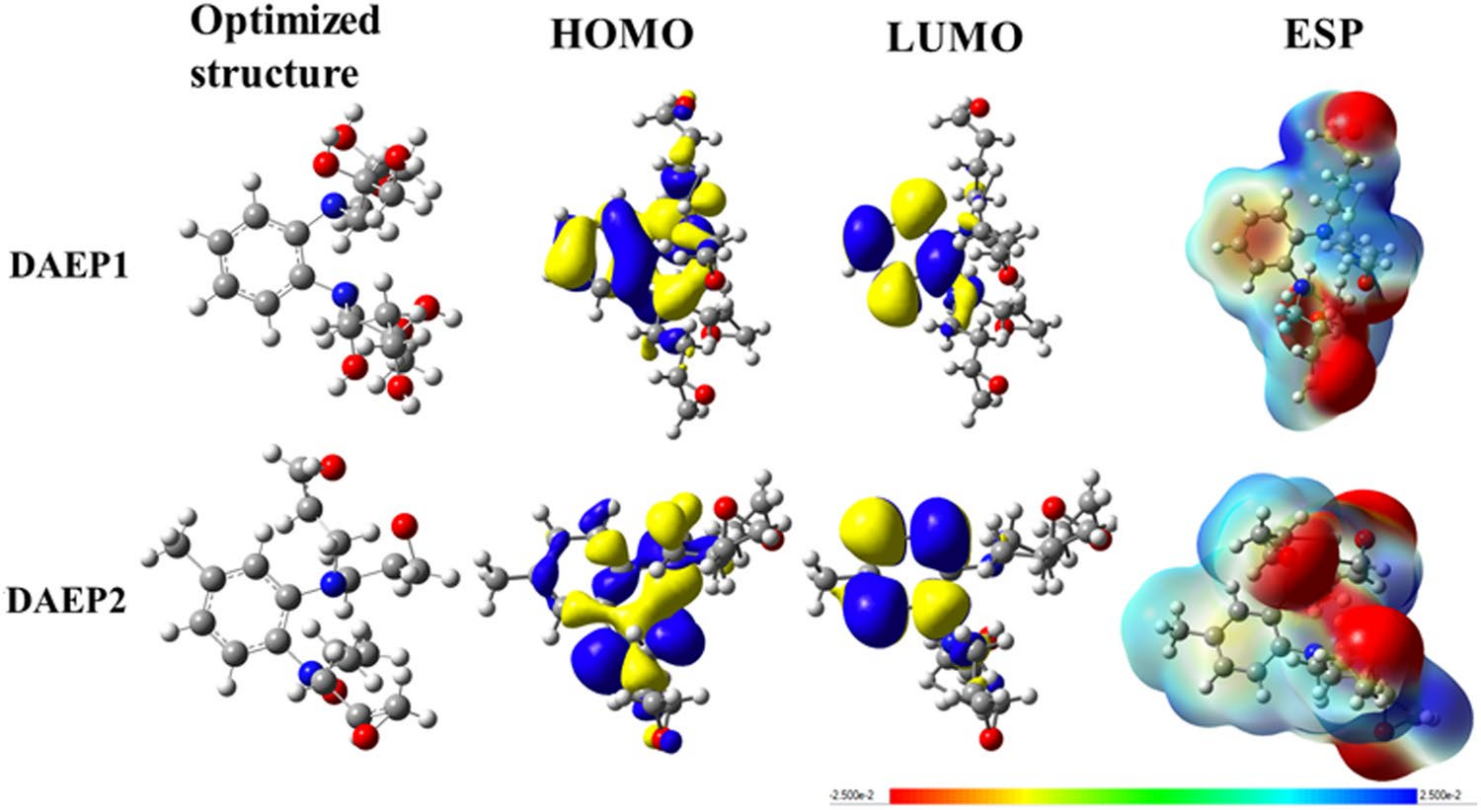
The optimized structures, HOMOs, LUMOs and ESP structures for theneutral inhibitor molecules using DFT/6-311 ++ G(d, p) calculation level in aqueous solution.

**Figure 9:**
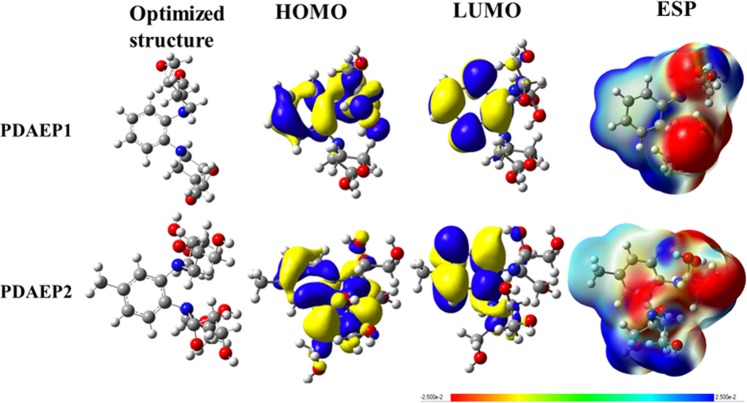
The optimized structures, HOMOs, LUMOs and ESP structures for the protonated inhibitor molecules using DFT/6-311 ++ G(**d, p**) calculation level in aqueous solution.

To validate the experimental results and to gain a deeper insight into the reactivity of the studied DAEP1 and DAEP2 compounds, the calculated quantum global parameters, which were extracted based on the values of the highest occupied and lowest unoccupied molecular orbitals (*E*_HOMO_ and *E*_LUMO_) were used. These parameters are the ionization potential (*I* = −*E*_HOMO_), the electron affinity (*A* = −*E*_LUMO_), the energy difference between *E*_LUMO_ and *E*_HOMO_, (ΔE = (*E*_HOMO_ − *E*_LUMO_), the electronegativity (*χ* = (I + A)/2) the global hardness (*η* = (I − A)/2) and softness (*σ* = 1/*η*), the fraction of electrons transferred (ΔN = (φ_Fe _− χ_inh_)/(2(η_Fe_ + η_inh_)), the electron transfer electron back-donation (ΔE_back-donation_ = −*η*/4) and the initial molecule-metal interaction energy (Δψ = ((χ_Fe_ − χ_inh_)^2^/(2(*η*_Fe_ + *η*_inh_)). It is important to mention that we selected work function(φ) is 4.82 eV for Fe (110) plan for the calculation of DFT parameters. The results are compiled in Table SI5, together with the total optimized energy (*E*)and the dipole moment (μ). Figures 8 and 9 show the optimized structures, the frontier molecular orbitals and electrostatic potential maps of the neutral and theprotonated species of the studied compound in aqueous solution, respectively. It was pointed out that the *E*_HOMO_ is frequentlyinterrelated with the electron donating propensity of the molecule into the vacant *d-*orbital of the metal, whereas, the *E*_LUMO_ corresponds to the electron acceptability of the inhibitor molecules from the vacant*d-*orbital of the metal surface^58–61^. The energy gap (ΔE) indicate the chemical reactivity of the inhibitorand its lower value associated with high efficiency^60–64^.

As can be observed from Fig. 9, that HOMO is mainly localized over phenyl ring, the amino groups and the epoxy oxygen whereas LUMO density is localized exclusively on the methylene carbon atoms of the phenyl ring, indicating that this is these regions are mainly involving in electron(s) donation and acceptation, respectively during the metal-inhibitors interactions. The observation was also supported by ESP map.

As indicated in Table SI5, *E*_HOMO_(DAEP2) > *E*_HOMO_ (DAEP1) in both gas phase and in aqueous solution. Similar trend is also seen for *E*_LUMO_ values, reflecting that DAEP2 moleculein its neutral form has the higher tendency to reactivity than DAEP1; this is not surprising considering that this inhibitor also has the higher tendency towards electron donation. Our obtained results show that the energy gap of DAEP2 in its neutral form is 0.105 and 0.113 eV smaller than that of the neutral DAEP2 in the gaps phase and aqueous solution, respectively. The solvent effect is clearly observing in decreasing of the energy gap of the studied species. Ongoing from gas phase to aqueous solution, our results show that the energy gap of DAEP2 decreases by 0.012 eV, which indicate that the DAEP2 compound is more reactive in aqueous solution than in the gas phase. The *η* and *σ* parameters are often correlated with the Pearson’s hard and soft acids and bases (HSAB) and the Lewis theory of acid and bases^61–67^. Our obtained results in Table SI5 show that the η value of DAEP2 is smaller than that of the DAEP1 molecule, and therefore, the softness quantity of the DAEP1 inhibitor is smaller than the DAEP2 compound. Similar results are also observed in aqueous solution. Obot and Gasem^68^ found that the compounds with the higher dipole moments are better inhibitors than those with lower dipole moments.

As indicated in Table SI5 that the dipole moment is aqueous solution of the DAEP2 is higher than the DAEP1, reflecting that the DAEP2 molecule is a more efficient inhibitor than the DAEP1 compound. It was pointed out as the number of electron transferred (ΔN)^68–70^ to the vacant or partially vacant d-orbitals of the metal increases the inhibition efficiency of the molecule increases^60,61,68^. As can be seen in Table SI5, the ΔN(110) obtained for the DAEP2 molecule is 0.041 and 0.030 higher than that obtained for the DAEP1 molecule in the gas phase and solution respectively, which suggests that the inhibition efficiency of the DAEP2 molecule is higher than that of the DAEP1 one. In addition, the other tabulated parameters such as χ, Δψ and ∆E_b-d_ support our conclusions, which validate the experimental results described in the experimental section of this study.

The frontier electron distribution for protonated form of DAEPs is shown Fig. 9 and it can be seen that frontier electron (HOMO and LUMO) distribution is similar as was observed in the case of the neutral species. Comparison between the protonated and neutral species is as follows:

- The values of *E*_HOMO_ and *E*_LUMO_ of the protonated species are higher than the corresponding ones of the neutral species.
- The protonated species are softer and less hardness than the neutral inhibitors.
- The fraction of electrons transferred (ΔN) of the protonated species to the vacant orbitals of the metal surface is found to be higher than that of the neutral species.
- The electron transfer electron back-donation energy (ΔE_back-donation_) is higher for the protonated inhibitors compared to the neutral ones.
- The initial molecule-metal interaction energy (Δψ) of the protonated inhibitors are higher than that of the neutral species.

As indicated in Table SI6 that the inhibition efficiency of the protonated inhibitors follows the order as: DAEP2 > DAEP1. Based on the above points, once can safely conclude that inhibition efficiency of the protonated species in aqueous solution is higher than in the gas phase.

#### MD simulation studies

**Figure 10:**
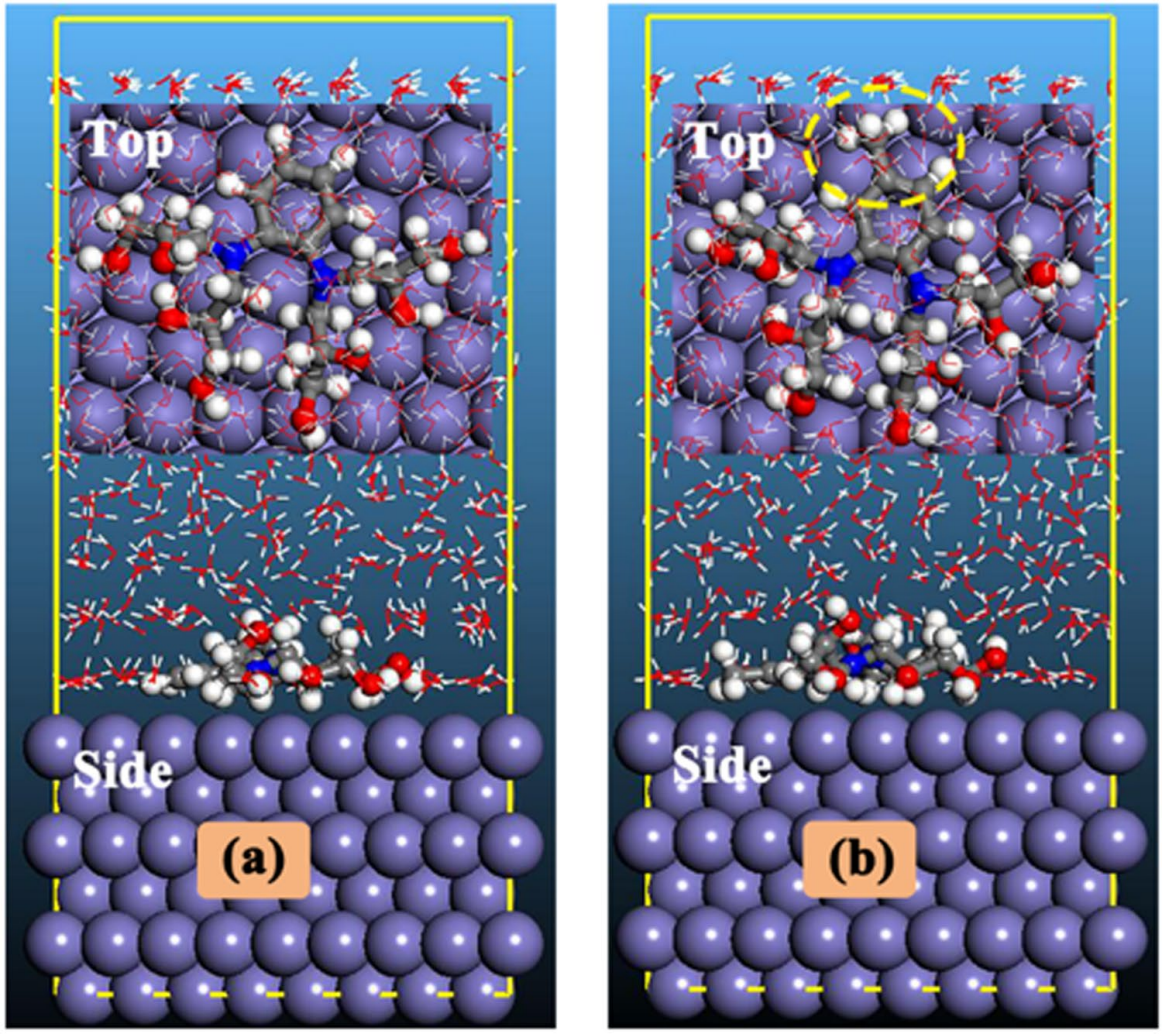
Side and top views of most stable adsorption configurations for (**a**) DAEP1 and (**b**) DAEP2 inhibitors on Fe(110) surface.

The nature of meta-DAEPs interactions and orientations of DAEPs on metallic surface was further studied using MD simulation method. The equilibrium configurations (top and side view) of inhibitors adsorbed on the Fe (110) surface are shown in Fig. 10. As can be seen from Fig. 10 that the adsorption centers of both inhibitors on the Fe(110) surface are the electrons of benzene rings, oxygen and nitrogen heteroatoms. The inhibitor molecules epoxy pre-polymers DAEP1and DAEP2 adsorbed almost flat orientation on the iron surface to maximize surface coverage and contact, ensuring a strong interaction for adsorbate/substrate system. The adsorption energy (*E*_ads_) for DAEPs adsorption can be calculated as follow (8):

8 $${E}_{ads}={E}_{total}-({E}_{surf+water}+{E}_{inh+water})+{E}_{water}$$

where *E*_total_ is denote the total energy related to metal-AEMs interactions, which include iron crystal, the adsorbed inhibitor molecule and solution; *E*_surf+water_ and *E*_inh+water_ are the potential energies of the system without the inhibitor and the system without the ironcrystal, respectively; *E*_water_ is the potential energy of the water molecules.

The obtained *E*_ads_ values are −834.1 and −853.3 kJ/mol for DAEP1 (a) and DAEP2 (b), respectively. Generally, the higher value of *E*_ads_ is consistent with the stronger the interaction between the inhibitors (DAEPs) and metal surface. Obviously, it appears that DAEP2 has a slightly higher absolute value of *E*_ads_ than DAEP1, and therefore it presents better inhibiting property for carbon steel, which is in consistent with the experimental findings.

**Figure 11:**
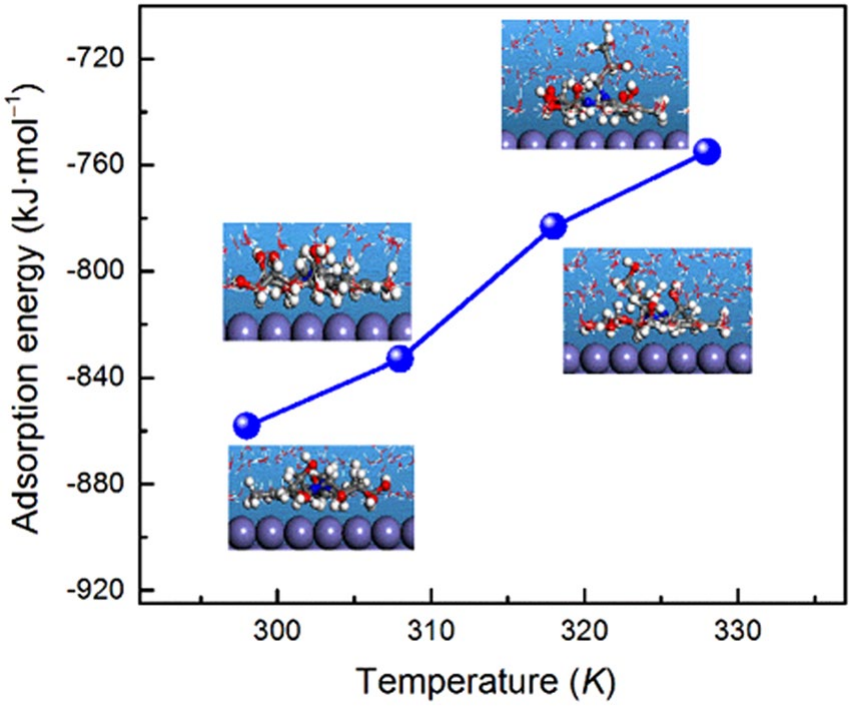
Dependence of adsorption energy on the temperature for adsorbed DAEP2 molecule on Fe(110).

The process of corrosion inhibition was highly influenced by the temperature. The equilibrium configurations of DAEP2 inhibitor adsorbed on the Fe(110) surface with different simulated temperatures were presented in Fig. 11. The obtained *E*_ads_ values are −853.3, −838.1, −780.5, and −754.7 kJ/mol at temperature 298 K, 308 K, 318 K and 328 K, respectively. From the results it can be observe that increase in the temperature causes significant decrease in the values of *E*_ads_ that can be resulted due to increase in the kinetic energy of the inhibitor molecules at elevated temperatures.

## Conclusions

Two diamine aromatic epoxy pre-polymers (DAEPs) namely, N^1^,N^1^,N^2^,N^2^-tetrakis (oxiran-2-ylmethyl) benzene-1,2-diamine (DAEP1) and 4-methyl-N^1^,N^1^,N^2^,N^2^-tetrakis (oxiran-2-ylmethyl) benzene-1,2-diamine (DAEP2) were synthesized, characterized and evaluated as corrosion inhibitors in the corrosion of carbon steel in acidic medium.

From above experimental and theoretical studies, it can be concluded that:

1. Both DAEP1 and DAEP2 act as good corrosion inhibitors and their inhibition effectiveness is concentration, temperature and substituent dependent. Obviously, high inhibition effectiveness was observed at low temperature, high inhibitor (DAEPs) concentration and electron releasing methyl substituent.
2. The highest inhibition efficiency values at 10^−3^ M (optimum concentration) are 91.7% and 92.9% for DAEP1 and DAEP2, respectively.

1. The PDP results reveal that, the diamine aromatic epoxy pre-polymers(DAEPs) behave as mixed type inhibitors.
2. EIS studies revealed the adsorption of DAEPs molecules and are confirmed by increase in *R*_ct_ and decrease in *C*_dl_ values, respectively.
3. Adsorption of the tested diamine aromatic epoxy pre-polymersmolecules obeyed Langmuir adsorption isotherm.
4. Very high negative magnitude of the ΔG_ads_ (−44.42 to −45.58 kJ/mol) values showed that DAEP1 and DAEP2 interact spontaneously and strongly with the metallic surface.
5. Theoretical results of the neutral and protonated species in both gas phase and aqueous solution indicated that the DAEP2 inhibitor is more efficient than DAEP1 one, which is completely agree with the experimental results.
6. The more negative value of *E*_ads_ for DAEP2 as compared to the DAEP1 indicates that later case has relatively strong probability of meta-inhibitor interactions as compared to former one.

## Supplementary information

Epoxy pre-polymers as new and effective materials for corrosion inhibition of carbon steel in acidic medium: Computational and experimental studies
