# Supplementary material for: Epoxy pre-polymers as new and effective materials for corrosion inhibition of carbon steel in acidic medium: Computational and experimental studies
Source: Sci Rep. 2019 Aug 12;9:11715. doi: 10.1038/s41598-019-48284-0 (PMC6691106; doi:10.1038/s41598-019-48284-0)
Supplement: Supplementary file 1 — Epoxy pre-polymers as new and effective materials for corrosion inhibition of carbon steel in acidic medium: Computational and experimental studies [file 41598_2019_48284_MOESM1_ESM.docx]

**SUPPLEMENTARY INFORMATIONS**

**Epoxy pre-polymers as new and effective materials for corrosion inhibition of carbon steel in acidic medium: Computational and experimental studies**

**Omar Dagdag^1^,Zaki Safi ^2^, Rachid Hsissou^1^, Hamid Erramli^3^, Mehdi El Bouchti^4^, NuhaWazzan^5^, Lei Guo^6^, ChandrabhanVerma^7,8*^, E. E. Ebenso^7,8*^ and Ahmed El Harfi^1^**

*^1^Laboratory of Agroresources, Polymers and Process Engineering (LAPPE), Department of Chemistry, Faculty of Science, IbnTofail University, BP 133, 14000 Kenitra, Morocco.*

*^2^Al Azhar University-Gaza, Chemistry Department, Faculty of Science, P.O Box 1277, Gaza, Palestine*

*^3^Laboratory of Materials, Electrochemistry and Environment, Department of Chemistry, Faculty of Sciences, IbnTofail University, Kenitra, Morocco.*

*^4^Higher School of Textile and Clothing Industries, Laboratory REMTEX, BP 7731, Oulfa, Casablanca, Morocco.*

*^5^King Abdulaziz University, Chemistry Department, Faculty of Science, P.O Box 42805, Jeddah, 21589, Saudi Arabia.*

*^6^School of Materials and Chemical Engineering, Tongren University, Tongren, 554300, China.*

*^7^Material Science Innovation & Modelling (MaSIM) Research Focus Area, Faculty of Natural and Agricultural Sciences, North-West University, Private Bag X2046, Mmabatho 2735, South Africa*

*^8^Department of Chemistry, Faculty of Natural and Agricultural Sciences, School of Chemical and Physical Sciences, North-West University, Private Bag X2046, Mmabatho 2735, South Africa.*

***Corresponding authors E-mails**: [chandraverma.rs.apc@itbhu.ac.in](mailto:chandraverma.rs.apc@itbhu.ac.in),[Eno.Ebenso@nwu.ac.za](mailto:Eno.Ebenso@nwu.ac.za)


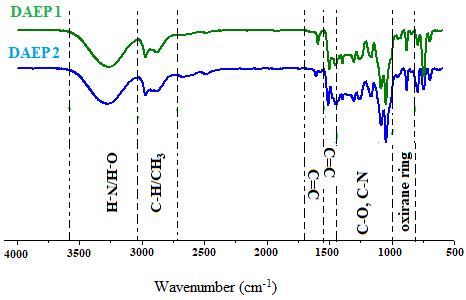


**Fig. SI 1.**ATR-FTIR spectra of DAEP1 and DAEP2


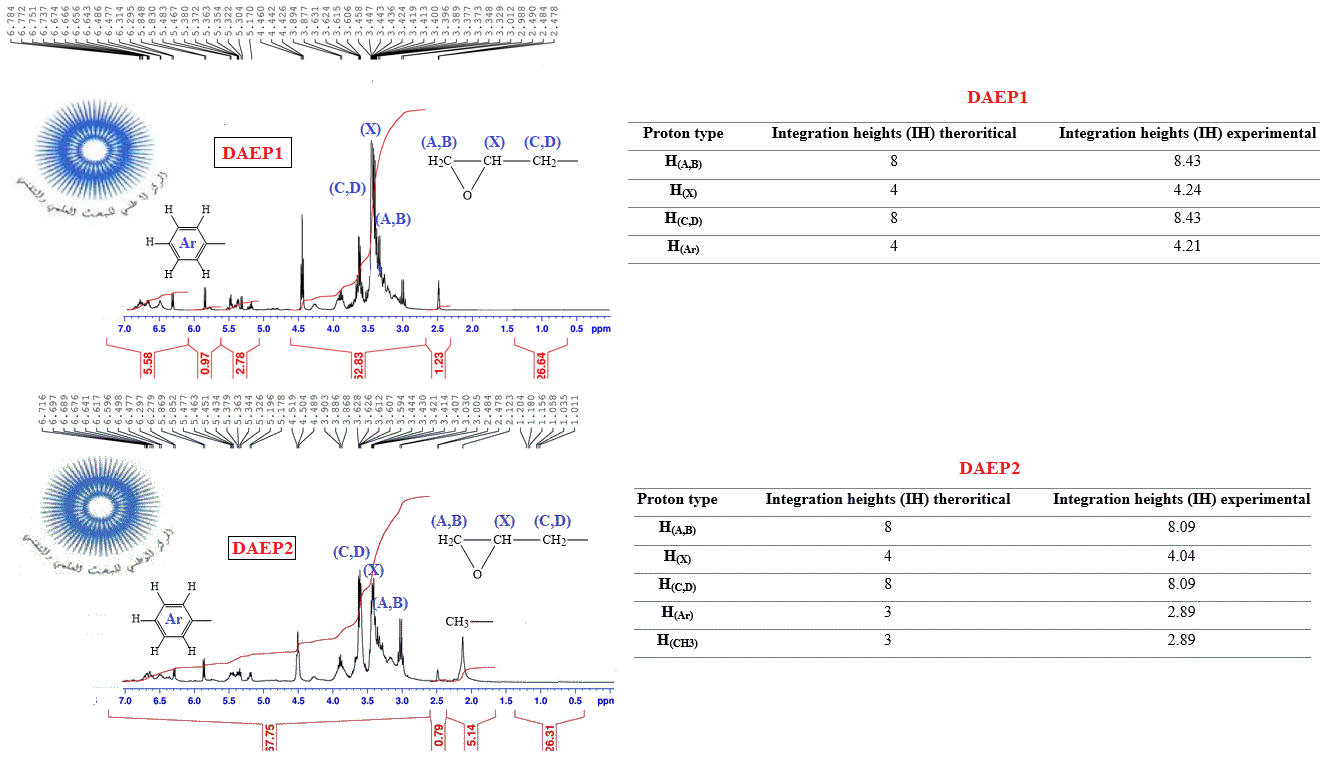


**Fig. SI 2.**^1^H NMR spectra of DAEP1 and DAEP2.


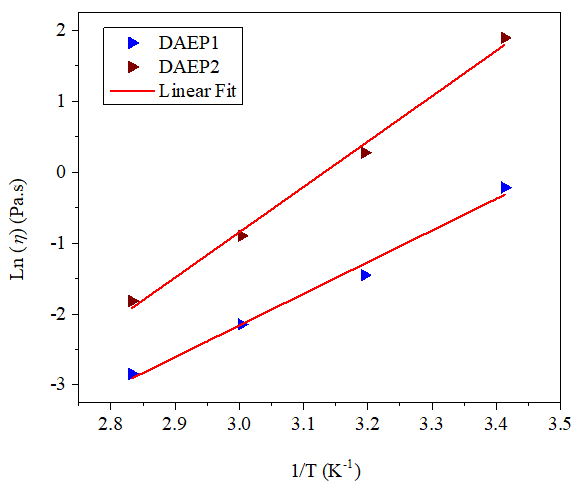


**Fig. SI 3.**The relationship between Ln (*η*)and 1/T for DAEP1 and DAEP2 at different temperatures.


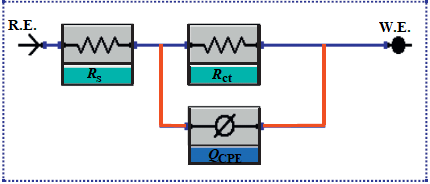


**Fig. SI 4.**Equivalent circuit used for the analysis ofthe EIS data.


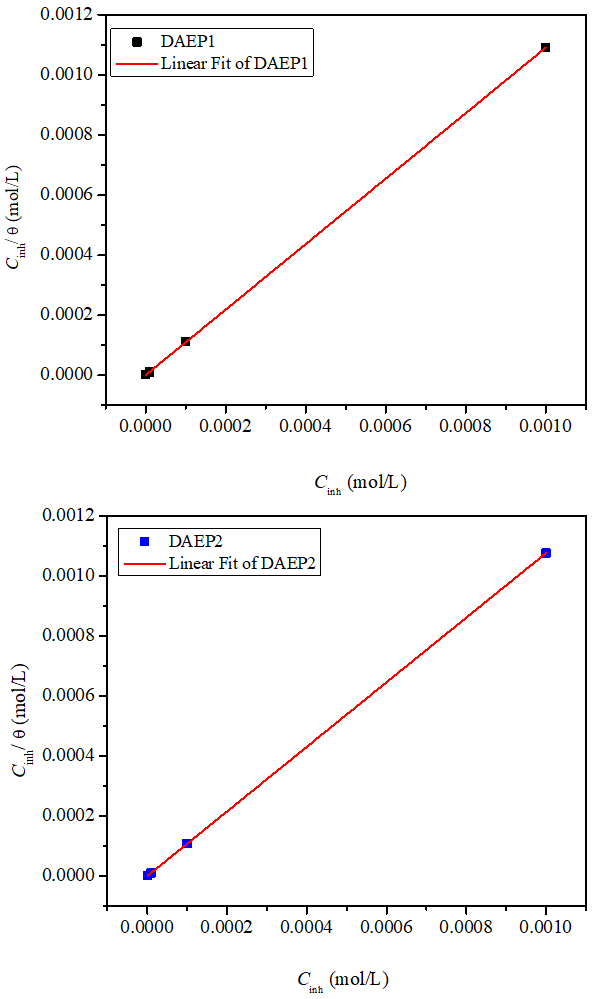


**Fig. SI 5.**Plot of the Langmuir adsorption isotherm of DAEP1 and DAEP2 on the carbon steel surface at 298 K.


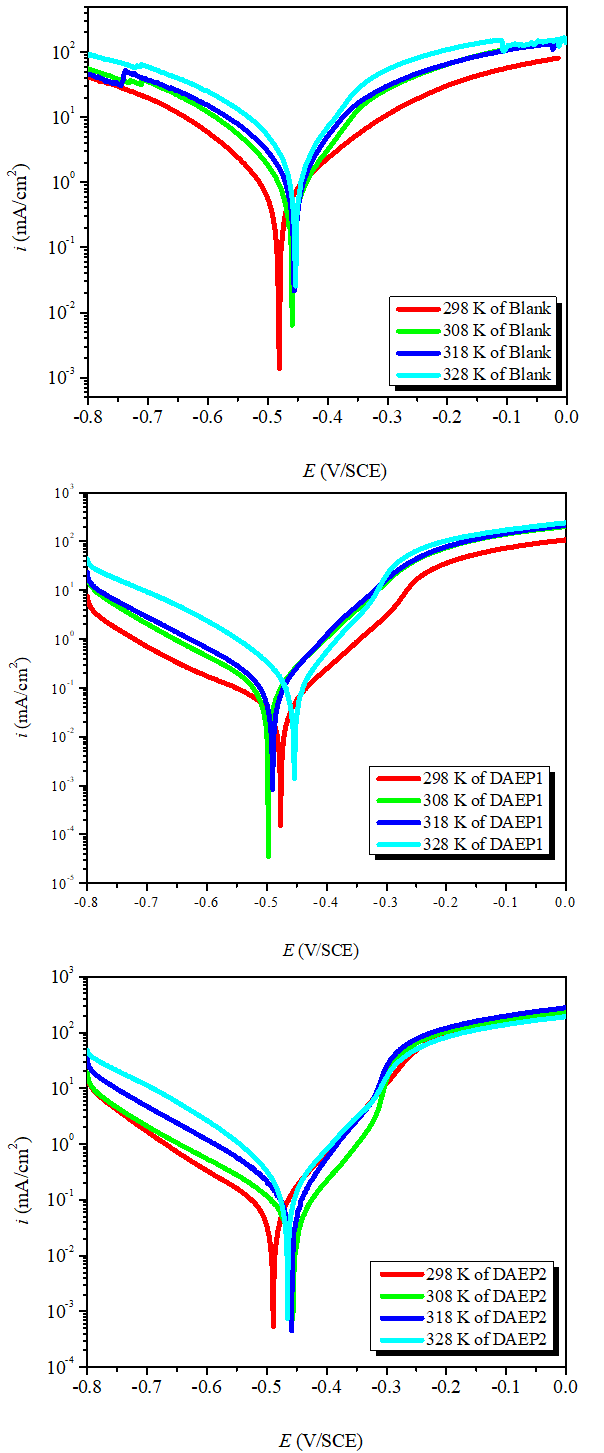


**Fig. SI 6.**Potentiodynamic polarization curves of carbon steel in 1 M HCl solution in the absence and presence of 10^-3^ M of DAEP1 and DAEP2 at different temperatures.

**Table**

**Table SI 1**Chemical structures, and abbreviations of the synthesized compounds.

| Abbreviation | Compounds structure | Yield/molar mass |
| --- | --- | --- |
| DAEP1  DAEP2 |  | Yield 90.5%  MM=472 g/mol  Yield 92%  MM=486 g/mol |

**Table SI 2**Analytical data of the synthesized compounds.

|  | ^1^H-NMR (ppm) (DMSO-d_6_, 300 MHz): | ATR-FTIR (cm^-1^) |
| --- | --- | --- |
| DAEP1 | The diamine aromatic epoxy pre-polymers DAEP1 showed signals at 6.39-6.61 ppm (s, 4H aromatic) (Ar) are attributed to the aromatic proton. The peak at 3.61 and 3.36 ppm (dd, 8H, N-CH_2_) (C,D) corresponds to the proton near to the oxirane ring. The spectrum shows characteristic peaks at 2.77 (m, 4H, CH oxirane ring) (X) and the peak at 2.63 and 2.38 ppm (dd, 8H, CH_2_) (A,B) ppm for the protons of the oxirane ring. | The ATR-FTIR spectrum of diamine aromatic epoxy pre-polymers DAEP1 shows a broad peak at 3268 cm^-1^ which could be attributed to stretching frequency of O–H or N–H presence in residual starting amine. The absorption bands at around 2970, 2918 and 2870 cm^-1^ corresponding to the stretching vibrations of C–H. The absorption peaks at 1584, 1500 and 1450 cm^-1^ reveal the presence of the aromatic ring. The bands within the range of 1000–1400 cm^−1^, which are attributed to the stretching vibrations of C–O and C–N, respectively. Two bands appear at 920 cm^-1^ and 835 cm^-1^ corresponding to the vibration of the oxirane ring. |
| DAEP2 | ^1^H-NMR for DAEP2 were appeared at 6.39-6.50 ppm (s, 3H aromatic) (Ar) are attributed to the aromatic proton. The peak at 3.61 and 3.36 ppm (dd, 8H, N-CH_2_) (C,D) corresponds to the proton near to the oxirane ring. The spectrum shows characteristic peaks at 2.77 (m, 4H, CH oxirane ring) (X) and the peak at 2.63 and 2.38 ppm (dd, 8H, CH_2_) (A,B) ppm for the protons of the oxirane ring. The peak at 2.34 ppm (s, 3H) (Methyl) is assigned to methyl proton, shifted downfield due to the electropositif effect of the group CH_3_. | The spectrum of DAEP2 shows a broad band at 3268 cm^−1^ which is a characteristic of the stretching vibration of O–H and N–H in residual starting amine. The bands observed in the 2970–2876 cm^-1^ region is characterized of C-H. The bands observed in wavelengths 1584, 1500 and 1450cm^-1^ are also characteristic of aromatic ring. Other peaks at 1299 and 1398 cm^-1^ are corresponding to the C-N vibration. Bands appeared in wavelengths of 1240, 1216 and 1089cm^-1^ showed then symmetric and asymmetric stretching vibrations of C-O band. The observation of a bands appear at 920 cm^-1^ and 835 cm^-1^ could be attributed to the oxirane groups. |

**Table SI 3**Langmuir adsorption parameters.

| Inh | *R*^2^ | slope | *K*_ads_ (M^-1^×10^6^) | $-\Delta G_{ads}$ (kJ.mol^-1^) |
| --- | --- | --- | --- | --- |
| DAEP1  DAEP2 | 0.9999  1.0000 | 1.0897  1.0758 | 1.10  1.50 | 44.42  45.18 |

**Table SI 4**The influence of temperature on the electrochemical parameters for carbon steel in 1 M HCl and 10^-3^ M of DAEP2.

| T (K) | *E*_corr_  mV/SCE | | *i*_corr_  µA/cm^2^ | | *η*% |
| --- | --- | --- | --- | --- | --- |
| 298  308  318  328 | **Blank**  - 473  - 459  - 455  - 453 | **10^-3^ M of DAEP1**  -489  -500  -491  -454 | **Blank**  916  1390  2700  4100 | **10^-3^ M of DAEP1**  075.40  160.45  419.16  923.82 | **-**  91.7  88.5  84.4  77.5 |
| 298  308  318  328 | **Blank**  - 473  - 459  - 455  - 453 | **10^-3^ M of DAEP2**  -489  -456  -458  -468 | **Blank**  916  1390  2700  4100 | **10^-3^ M of DAEP2**  070.52  120.00  250.59  501.86 | **-**  92.3  91.3  90.7  87.7 |

Table SI 5Quantum chemical parameters for the neutral forms of DAEP1 and DAEP2 in both gas phase and aqueous solution, calculated using B3LYP/ 6-311++G(d,p).

|  | Gas phase | |  | | Aqueous solution | |
| --- | --- | --- | --- | --- | --- | --- |
|  | DAEP1 | DAEP2 |  | | DAEP1 | DAEP2 |
|  | Neutral form | | | | | |
| *E*(hr) | -1110.6142 | -1149.9384 |  | -1110.632 | | -1149.956 |
| *μ*(Debye) | 6.50 | 8.50 |  | 3.49 | | 4.36 |
| *E*_HOMO_(eV) | -5.669 | -5.443 |  | -5.570 | | -5.405 |
| *E*_LUMO_(eV) | -0.565 | -0.444 |  | -0.470 | | -0.418 |
| ΔE (eV) | 5.104 | 5.126 |  | 4.973 | | 4.987 |
| I (eV) | 5.669 | 5.443 |  | 5.570 | | 5.405 |
| A (eV) | 0.565 | 0.444 |  | 0.470 | | 0.418 |
| χ (eV) | 3.117 | 2.943 |  | 3.020 | | 2.912 |
| η (eV) | 2.552 | 2.500 |  | 2.550 | | 2.493 |
| σ (eV^-1^) | 0.392 | 0.400 |  | 0.392 | | 0.401 |
| ΔN110 | 0.334 | 0.375 |  | 0.353 | | 0.383 |
| Δψ (eV) | 1.477 | 1.646 |  | 1.553 | | 1.676 |
| ∆E_b-d_ (eV) | -0.638 | -0.625 |  | -0.638 | | -0.623 |

Table SI 6Quantum chemical parameters for the protonated species of DAEP1 and DAEP2 in both gas phase and aqueous solution, calculated using B3LYP/ 6-311++G(d,p).

|  | Gas phase | |  | Aqueous solution | |
| --- | --- | --- | --- | --- | --- |
|  | DAEP1 | DAEP2 |  | DAEP1 | DAEP2 |
| *E*(hr) | -1416.5046 | -1416.5351 |  | -1455.8250 | -1455.8515 |
| *μ*(Debye) | 7.81 | 10.20 |  | 7.46 | 10.78 |
| *E*_HOMO_(eV) | -5.104 | -5.049 |  | -5.360 | -5.222 |
| *E*_LUMO_(eV) | -0.481 | -0.466 |  | -0.350 | -0.241 |
| ΔE (eV) | 4.623 | 5.011 |  | 4.583 | 4.981 |
| I (eV) | 5.104 | 5.049 |  | 5.360 | 5.222 |
| A (eV) | 0.481 | 0.466 |  | 0.350 | 0.241 |
| χ (eV) | 2.792 | 2.757 |  | 2.855 | 2.731 |
| *η* (eV) | 2.312 | 2.291 |  | 2.505 | 2.491 |
| *σ* (eV^-1^) | 0.433 | 0.436 |  | 0.399 | 0.402 |
| ΔN(110) | 0.439 | 0.450 |  | 0.392 | 0.419 |
| Δψ (eV) | 1.915 | 1.964 |  | 1.714 | 1.829 |
| ∆E_b-d_ (eV) | -0.578 | -0.573 |  | -0.626 | -0.623 |
